# Neural Signature and Decoding of Unmanned Aerial Vehicle Operators in Emergency Scenarios Using Electroencephalography

**DOI:** 10.3390/s24196304

**Published:** 2024-09-29

**Authors:** Manyu Liu, Ying Liu, Aberham Genetu Feleke, Weijie Fei, Luzheng Bi

**Affiliations:** School of Mechanical Engineering, Beijing Institute of Technology, Beijing 100081, China; lusuammy@outlook.com (M.L.); biggirlliu@bit.edu.cn (Y.L.); 7620220003@bit.edu.cn (A.G.F.); bhxblz@bit.edu.cn (L.B.)

**Keywords:** electroencephalogram, brain–computer interface, emergency detection, brain neural signature

## Abstract

Brain–computer interface (BCI) offers a novel means of communication and control for individuals with disabilities and can also enhance the interactions between humans and machines for the broader population. This paper explores the brain neural signatures of unmanned aerial vehicle (UAV) operators in emergencies and develops an operator’s electroencephalography (EEG) signals-based detection method for UAV emergencies. We found regularity characteristics similar to classic event-related potential (ERP) components like visual mismatch negativity (vMMN) and contingent negative variation (CNV). Source analysis revealed a sequential activation of the occipital, temporal, and frontal lobes following the onset of emergencies, corresponding to the processing of attention, emotion, and motor intention triggered by visual stimuli. Furthermore, an online detection system was implemented and tested. Experimental results showed that the system achieved an average accuracy of over 88% in detecting emergencies with a detection latency of 431.95 ms from the emergency onset. This work lays a foundation for understanding the brain activities of operators in emergencies and developing an EEG-based detection method for emergencies to assist UAV operations.

## 1. Introduction

Brain-computer interface (BCI) is a direct communication pathway between the brain and an external device, enabling users to control computer systems or other devices through neural signals without the need for muscle activity [1,2]. Electroencephalography (EEG) stands out as a prevalent method for acquiring neural signals in BCI systems because of its non-invasive nature, high temporal resolution, and relatively low cost [3,4].

BCI offers a novel means of communication and control for individuals with disabilities [5,6,7] and can also enhance the interactions between humans and machines for the broader population [8,9]. In recent years, BCI has seen widespread application across various domains, such as medical rehabilitation [7,10], gaming [11,12], military training [13], psychology [14,15], and education [16,17]. The application of BCIs in human state detection is particularly noteworthy. Numerous studies have demonstrated that BCI systems can identify states like attention [18], emotion [19], and fatigue [20], thus paving the way for personalized services and health monitoring. Recently, EEG-based state monitoring has also been applied to detect emergencies, and the brain activity of drivers in emergencies is gradually being revealed. In 2011, Haufe et al. [21] studied neural correlation and decoding of driver emergency response using EEG signals. They discovered event-related potentials (ERPs) related to motor preparation, confirming the feasibility of using EEG to detect drivers’ driving responses in emergencies. In 2015, Kim et al. [22] distinguished emergency braking caused by emergencies and soft braking caused by drivers’ normal braking intention based on ERP features and combined features, showing that the classifier based on combined features can detect drivers’ responses faster than the classifier based on ERP features. In 2018, Wang et al. [23] established an EEG-based detection model of driver emergency responses, which integrated the results of the proposed EEG-based intention detection model with the results of the obstacle detection model based on environmental information to achieve the recognition of emergencies.

Like drivers’ emergency response detection of ground vehicles, operators’ emergency response detection of unmanned aerial vehicles (UAVs) is crucial to better assist operators in addressing these emergencies [24,25]. Furthermore, the brain activities of a drone operator in emergencies differ from those of a vehicle driver because vehicles travel faster, and emergencies are more significant stimuli to drivers.

Thus, it is necessary to explore neural signatures and corresponding decoding methods of UAV operators under emergencies. However, to our knowledge, no studies focus on this issue, although some researchers investigated how to apply a BCI to control a drone [26,27,28,29].

In this paper, we investigate the neural signatures of brain activity in UAV operators during emergencies and propose a novel method for detecting their emergency responses using EEG signals. Additionally, we establish an online testing system to rigorously evaluate the performance of this method. The primary contribution of this work is twofold. Firstly, it represents the first comprehensive study of UAV operators’ brain activity in emergency situations, highlighting specific neural signatures associated with their responses. Secondly, we develop an EEG-based detection system that is able to identify these emergency responses with over 88% accuracy and a response time of 431.95 ms. This work lays a foundation for understanding the brain activities of operators in emergencies and developing an EEG-based detection method for emergencies to assist UAV operations.

## 2. Materials and Methods

### 2.1. Experimental Paradigm and Procedure

The experiments involved eight healthy participants, aged 22 to 24, with a balanced gender distribution. They all had normal vision and were confirmed to have no brain diseases. This study was conducted at the Beijing Institute of Technology, Beijing, China, and was approved by the Local Ethics Committee of the Beijing Institute of Technology (approval number: BIT-EC-H-2023016). All subjects were informed of the consent form before the experiment, and the experiment adhered to the Declaration of Helsinki.

To simulate the UAV’s flight in a complex environment, we utilized real videos of UAV flights captured in underground parking lots. The footage was captured using the UAV’s integrated front-facing camera, which simulated the visual perspective experienced during actual flights. Before the experiment, the videos were segmented into 12 non-emergency and 12 emergency videos. Each emergency video was cropped five seconds before the drone hit a different obstacle. Obstacles included walls, pillars, indicators, fire hydrants, and other common objects in underground parking lots. All subjects were asked to use a small handheld keyboard to simulate the maneuvering of a UAV.

**Figure 1:**
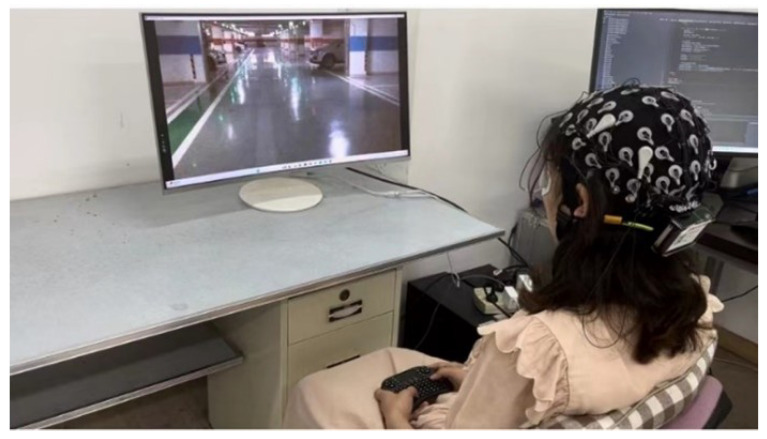
Experiment setup.

**Figure 2:**
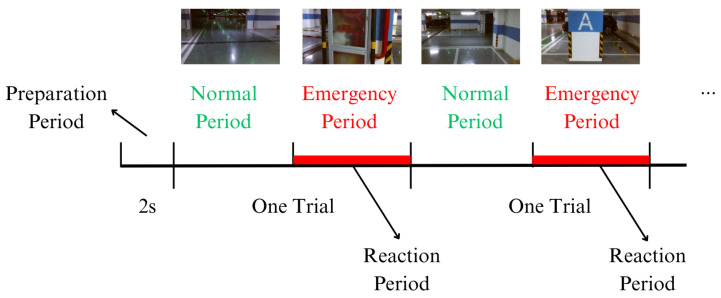
Experimental paradigm.

Figure 1 and Figure 2 illustrate the experimental setup and procedure. Each experiment was composed of 20 runs with a rest period of 2 min in between. Each run consisted of 12 trials. In each trial, a non-emergency video appeared, followed by an emergency video, and each video was played without an interval. Video sequences were not repeated within a single experimental session. Each trial was played without an interval. In each trial, subjects were asked to press the handheld keyboard when they judged the UAV was about to install obstacles and needed to avoid obstacles. Because the instantaneous switch of the stimuli might also cause changes in brain activity, the appearance of both kinds of videos was used as an event for subsequent analysis. In this paper, the two kinds of events were defined as non-emergency and emergency events, respectively.

### 2.2. Data Collection and Processing

EEG signals were acquired by a 64-electrode portable wireless EEG amplifier (NeuSen. W64, Neuracle, China) from the scalp of subjects at the Fpz, Fp1, Fp2, AF3, AF4, Fz, F1, F2, F3, F4, F5, F6, FCz, FC1, FC2, FC3, FC4, Cz, C1, C2, C3, C4, T7, T8, CP1, CP2, TP7, TP8, Pz, P3, P4, P7, P8, POz, Oz, O1, O2 locations according to an international 10–20 system, with a forehead ground at AFz and reference placed at CPz. Electrooculogram (EOG) signals were acquired from two electrodes positioned below the outer canthi of the eyes. The sampling rate was set to be 1000 Hz. Electrode impedances were calibrated to be less than 5 KΩ.

In this paper, the processing procedure included downsampling, baseline correction, independent component analysis (ICA), artifact subspace reconstruction (ASR), and band-pass filtering. To reduce the amount of data, the raw EEG data were downsampled to 200 Hz. Then, the data were corrected at baseline, using the average of the top 100 data points for each data point. The FastICA package and asrpy package were used in the next preprocessing. For the ERP detection, a fourth-order [0.1–5] Hz band-pass Butterworth filter was used to reserve the low-frequency component of EEG signals.

### 2.3. Neural Signatures

After removing samples with a maximum value of more than 100 µV, the remaining samples were prepared for analysis. To compare the brain activity patterns of drone operators in non-emergency and emergency situations, we extracted data segments of [−2, 2] s relative to the onset of both emergency and non-emergency events. In total, 240 samples were extracted for each subject in each situation.

**(1) ERPs:** To explore the regularity of subjects’ brain activity and visualize the ERPs, we preprocessed the selected EEG data, superimposed and averaged the sample data of all subjects, and plotted the time-domain amplitude. Visual mismatch negativity (vMMN) is a phenomenon that reflects the brain’s automatic comparison between current and previous visual stimuli [30]. It can also be interpreted as the difference between the current stimulus and expectations based on previous information [31]. It is usually found at electrode locations over the posterior scalp [32]. Thus, we selected the POz channel to observe the vMMN. The contingent negative variation (CNV) is associated with attention allocation in anticipation of an imperative stimulus to prepare a subsequent behavioral response with its peak exhibited around stimulus onset over the prefrontal cortex [33], which may reflect response caution adjustments in perceptual decision-making and more negative CNV amplitudes are associated with shorter response times [34]. In this paper, we selected the Fz channel to visualize the CNV.

**(2) Source Analysis:** EEG data were transformed from the sensor to the source level by source imaging techniques to visualize brain activation patterns in the source space. A forward head model was initially constructed to ascertain the propagation patterns of electrical fields from the cortex to the scalp. This model was developed by co-registering the ICBM152 boundary element model (BEM) with the EEG electrodes using the open-source software Brainstorm 3.4. The conductivities assigned to the scalp, skull, and brain layers of the BEM were 1, 0.0125, and 1, respectively. The forward model was then estimated using OpenMEEG BEM in the source space of the cortex surface. Subsequently, the inverse estimation of brain sources was computed employing sLORETA with unconstrained dipole orientations and the minimum norm imaging method.

### 2.4. Decoding Models

In this part, a 5-fold cross-validation with 10 cycles was employed to assess the performance of the classification model. Both the training and testing sets utilized the same preprocessing procedures detailed above, including downsampling, baseline correction, removal of eye artifacts using ICA, motion artifact removal through ASR, and filtering within the 0.1–5 Hz frequency range. The ICA unmixing matrix obtained from the training set was directly applied to the testing set. After this, taking the onset of the emergencies as the zero point, we extracted the data of [−3, −2.5] s as normal samples and the data of [−0.25, 0.25] s as emergency samples.

We used hierarchical discriminant component analysis (HDCA) for feature extraction and classification, which is the classic method in target detection [35]. The principle underlying HDCA assumes that the spatial activity distribution of EEG varies over time, and the sample data can be segmented into several consecutive N ms windows with a time resolution of N ms. Then, it can find the weight vector of each window. Based on this principle, linear discriminant analysis (LDA) was used for the spatiotemporal feature extraction in this paper.

First, the EEG signals of each channel were evenly divided into four segments according to a 125 ms time window. Then, LDA was used to calculate the best weight vector of each window to maximize the difference between the EEG signals of emergency and normal samples. Therefore, multichannel EEG signals were compressed into single-channel signals, as follows.

(1) $$y_{n}=\sum_{i=1}^{ch}w_{i,n}x_{i,n}$$

where ch means the the number of channels, *i* and *n* represent the indices of channels and segmented windows, respectively. $x_{i,n}$ and $w_{n,i}$ represent the EEG signal and weight of the *i* channel and *n* segment, respectively. $y_{n}$ means the single channel signal.

**Figure 3:**
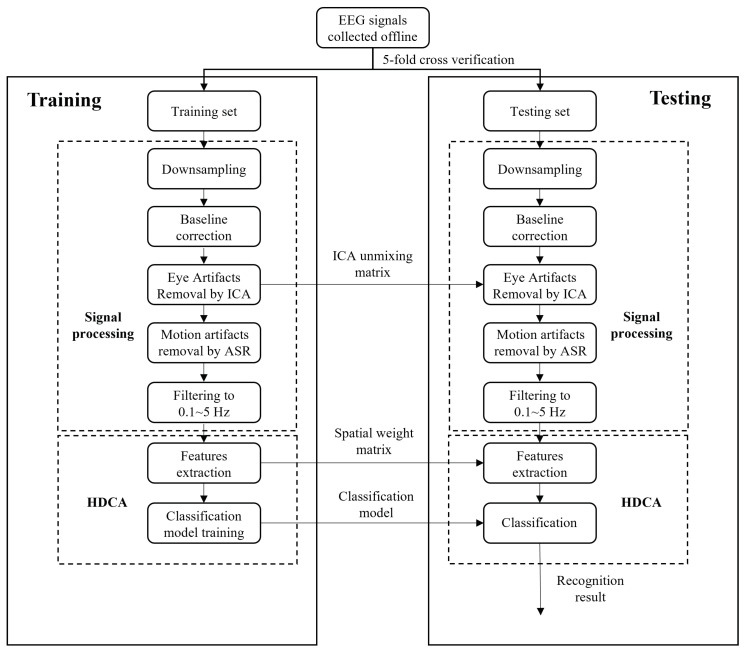
Schematic diagram of classification.

Then, another LDA classifier was used to classify $y_{n}$ into the final result. Note that the $w_{n,i}$ obtained in the train datasets was used in the test datasets directly. The complete schematic diagram of classification is shown in Figure 3.

In addition to HDCA mentioned above, LDA, SVM, XGBoost, and EEGNet were used for comparison.

### 2.5. Online Detection System

**Figure 4:**
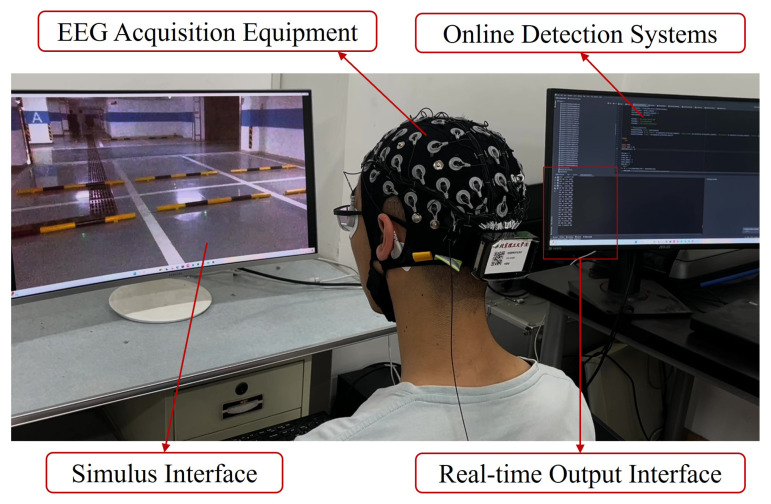
Online detection system.

In this paper, an online detection system was set up to test the applicability of detecting emergencies using EEG. Figure 4 shows the components of the online detection system. It mainly consisted of four parts: the stimulus interface, the EEG acquisition part, the online decoding part, and the real-time output interface. The model was trained before the whole experiment. In the experimental setup, the online detection and result output components were managed by the primary thread, and the stimulation interface and EEG data acquisition were delegated to two subordinate threads. An interface was configured to facilitate seamless data exchange among the two subordinate and primary threads.

**Figure 5:**
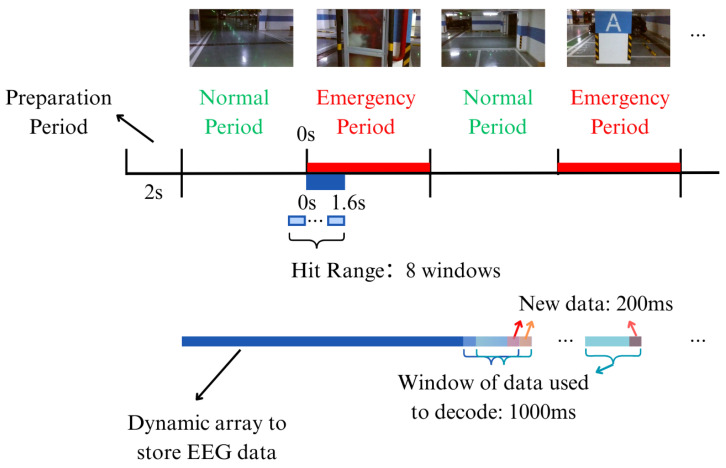
Paradigm of online detecting experiment. It shows how the EEG data are stored and how the data are intercepted for decoding. The hit range contains eight windows, and if any two consecutive windows in these eight windows are decoded as emergency, it is considered as being hit correctly.

**Figure 6:**
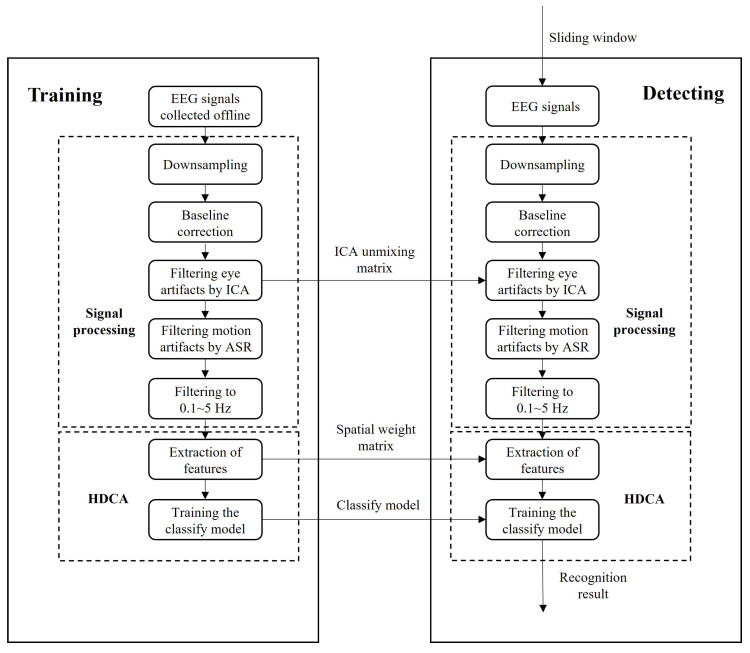
Principle of online detection system.

The online detection system was similar to the offline classification system, comprising the following steps: first, real-time EEG data acquisition was performed using the same electrode configuration as in the offline system. Next, the acquired data underwent preprocessing, which included downsampling, baseline correction, removal of eye artifacts using ICA, motion artifact removal through ASR, and filtering within the 0.1–5 Hz frequency range. However, the difference was that the whole set of samples taken from data collected offline was the training set, whereas the data collected online were used for detection. As shown in Figure 5, the EEG data collection was a continuous process where each new segment of data was added in real time to a sequentially expanding array. This array acted as a cumulative repository for the EEG signals, storing them as they were received. The system periodically extracted the most recent 1-second segment of EEG data from the end of this array every 200 ms for the subsequent detection. The principle of the detection system is shown in Figure 6, and the pseudocode is shown in Algorithm 1. Consequently, the online detection system generated a command every 200 ms. If two successive commands indicated an emergency, the system determined that an emergency situation was detected.
**Algorithm 1** EEG Signal Processing and Experiment Execution **Input:** EEG signal $X_{n}$, where $X_{n}\in R^{C\times S},n=1,\ldots ,N$; Selected channel index $C_{i},i=1,\ldots ,ch$; Trained prediction model f(X) ICA matrix of train datasets ω **Output:** Prediction results *y*1:Obtain selected EEG $X_{1}$ using channel index $C_{i}$ from $X_{n}$2:Apply base sliding correction to $X_{1}$ to get corrected data $X_{2}$3:Remove eye artifacts from $X_{2}$ using ICA matrix ω to get data $X_{3}$4:Apply ASR to $X_{3}$ to get clean data $X_{4}$5:Apply a Butterworth filter to $X_{4}$ to get processed data $X_{n}^{\prime }$, where $X_{n}^{\prime }\in R^{ch\times S},n=1,\ldots ,ch$;6:Get prediction results using model $y=f(X_{n}^{\prime })$

Similarly, eight subjects participated in the online detection experiments. Before the experiments, the subjects were asked to perform 10 runs of the same experiments for offline data collection, and the data collected offline were used for model training. Every two runs, the subjects were asked to rest for 2 min. After the offline data collection, the subjects rested for 10 min, and then 10 runs of online experiments were performed. Every two runs, the subjects were asked to rest for 2 min, too. The detection results were displayed on the screen in real time. After one run, the detection indicators were printed on the screen. All of the detection results were recorded for the evaluation of the subsequent average detection indexes.

In this paper, detection indexes of online experiments included recall, FAR (false alarm rate), accuracy, and response time. Recall is defined as the percentage of the experiments that correctly hit in all experiments and hitting correctly means the system detects emergency within 1 s of the emergency event appearance. FAR is defined as the percentage of the error emergency instructions outputting in normal stages in all instructions in normal stages. Accuracy is a comprehensive index of system performance evaluation, which can be calculated through the following formula.

(2) $$Accuracy=\frac{Recall+(1-FAR)}{2}$$

## 3. Result and Discussion

### 3.1. Neural Signatures

**Figure 7:**
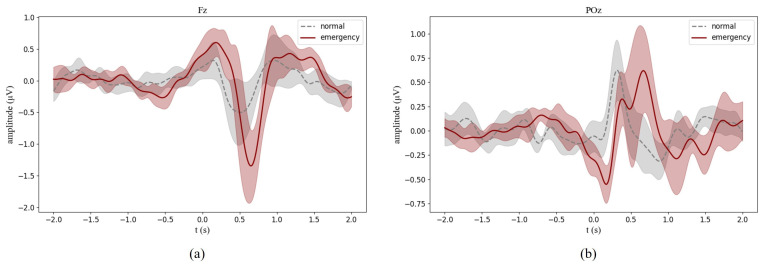
Average ERPs of all subjects. Valid data of all trials and all subjects are superimposed and averaged. For the solid line, time 0 is the time point when the emergency event appears, and for the dotted line, time 0 is the time point when the non-emergency event appears. (**a**,**b**) represent the ERPs at electrodes Fz and POz, respectively.

Figure 7 shows the average ERPs at the electrodes Fz and Poz across all subjects. For the solid line, time 0 s was the time point when the emergency events appear, and for the dotted line, time 0 s was the time point when the non-emergency event appears. By comparison, although there was a clear spike in both cases, the trend of change in the solid line was more obvious, and there were unique phenomena in the solid line. At the electrode POz, there was a drastic negative offset, which peaked negatively at around 0.2 s in the solid line. There was a positive offset at the electrode POz, and a negative offset after 0 s at the electrode Fz after 0 s in both cases. We applied the Wilcoxon rank sum test to explore whether there were significant differences in neural characteristics of brain activities between the two cases. The positive peak of the two lines was not statistically different at the electrode Poz (0.72 µV vs. 0.84 µV, *p* = 0.46 > 0.05). However, the negative peak value of Fz in the solid line was larger than in the dotted line, and it was statistically significant (−0.70 µV vs. −1.61 µV, *p* = 0.015 < 0.05). The negative peak was statistically different at Poz (−0.43 µV vs. 0.70 µV, *p* = 0.039 < 0.05). Thus, in this part, the negative peaks were used for the following analysis.

The significant difference in the ERPs between the two cases meant that emergencies produced significantly different brain activities. In the emergencies, the negative peak of POz coincided with the classical ERP components N2, possibly caused by the vMMN. In the experiment, when emergencies occurred, the previous normal state could be considered the preceding memory, and the emergencies could be understood as an event that did not match the memory. Notably, almost no significant negative shift was induced by the non-emergency event. Some researchers consider that the negative emotion-related vMMN effect is significantly greater than the neutral-related one [36,37,38]. In the event of emergencies, subjects might have negative emotions like nervousness. Thus, it was reasonable that more pronounced vMMN occurred in emergencies. The positive peak at POz in both states might be a component similar to P300, reflecting the switching of the footage. Affected by the previous negative peak, the positive peak might be put off in emergencies. The negative peak at Fz might coincide with the ERP components CNV. CNV reflects the stage of preparation for a response after a stimulus occurs, and the value of the peak correlates with the urgency of the response [34]. In the experiment, the negative peak at Fz might reflect the brain’s cognitive processing of whether to respond immediately or not. When the emergencies occurred, the subject needed to react immediately, so a more urgent response might lead to a larger negative peak. The negative peak appeared at about 0.2 s at POz and about 0.6 s at Fz, consistent with the sequence of brain activities. The subjects first received visual stimulation, processed visual information, and then performed cognitive processing. The ERPs of individuals are listed in Appendix A, which shows that the pattern explored above is universal across the eight subjects, although there are slight differences between subjects.

**Figure 8:**
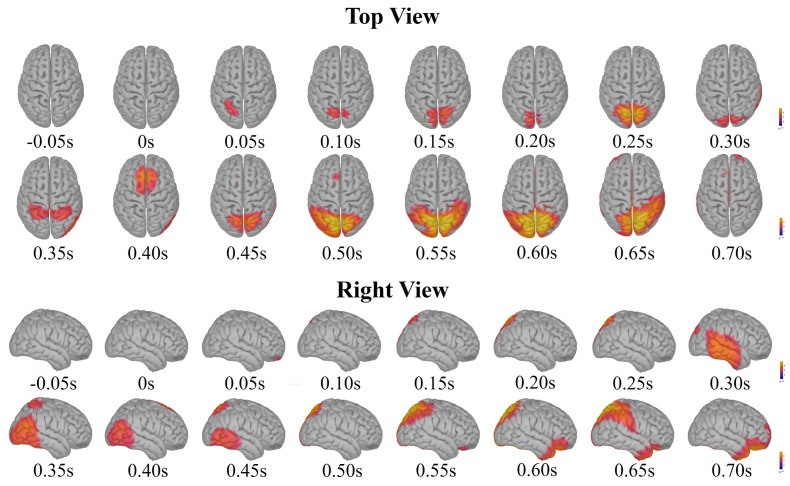
The grand-averaged activation pattern of one subject in the source space. The source imaging results are displayed from time lags −100 ms to 700 ms with a time interval of 50 ms. The source-space activity is imaged from two projection angles: top view and right view.

Figure 8 shows the grand-averaged activation pattern in the source space of one subject, and those of the rest subjects are displayed in Appendix B. According to the source analysis results of one subject in Figure 8, within 0.2 s to 0.4 s of the onset of emergencies, the occipital area of the brain was activated, and then the parietal, temporal, and frontal areas were sequentially activated, confirming that the brain response was generated by visual stimulation. When the emergencies occurr, the subjects might have a certain tension, and then through cognitive processing and decision-making, take the action of pressing the key. Thus, the phenomenon of the sequential activation of the brain areas might be related to emotional processing, cognitive processing, and decision-making. After 0.45 s, the occipital lobe sustained the intense activation, which might be related to the stronger visual stimulation of the drone about to hit an obstacle. However, as shown in Appendix B, the outcomes of source analysis varied across subjects. Not all three brain regions were sequentially activated within the source space for every individual, suggesting that individuals’ resulting emotional and behavioral responses are inherently diverse. This variability might stem from the individual differences in cognitive information processing. Furthermore, the temporal dynamics of brain activation were subject-specific, reflecting the inherent differences in individuals’ reaction time.

### 3.2. Classification Performance

**Figure 9:**
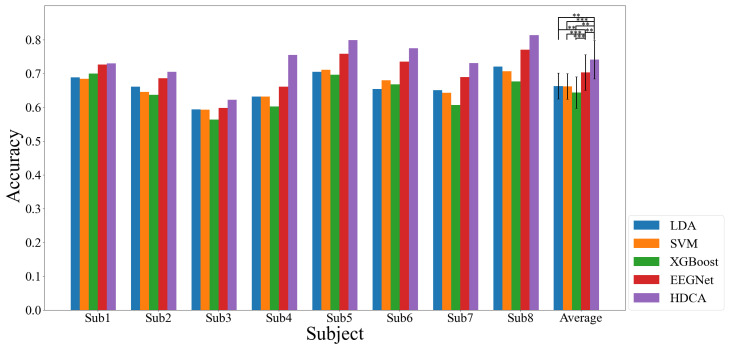
The performance comparison of five methods for each individual subject. The error bar means the standard deviation of eight subjects, and the asterisk represents the results of the significance analysis (paired *t*-test: ** *p* < 0.01, *** *p* < 0.001).

The efficacy of the classification models was meticulously assessed through a rigorous offline data-evaluation process. The models’ performance was gauged using a five-fold cross-validation methodology, which was executed ten times to ensure the robustness and reliability of the findings. We tested statistical differences between the five methods with the permutation-paired *t*-tests and controlled the false discovery rate (FDR). Figure 9 shows the accuracy comparison of the five models. As shown in Figure 9, the accuracy significantly exceeded the chance level, validating the hypothesis that EEG signals from drone operators could be effectively distinguished between normal operating and emergency scenarios.

Furthermore, HDCA performed best (HDCA vs. LDA, paired *t*-test, *p* = 0.0010 < 0.01; HDCA vs. SVM, paired *t*-test, *p* = 0.0008 < 0.001; HDCA vs. XGBoost, paired *t*-test, *p* = 0.0008 < 0.001; HDCA vs. EEGNet, paired *t*-test, *p* = 0.0072 < 0.01). Thus, HDCA was selected to develop the online detection system. The classification results of HDCA are reported in Table 1. It was found that the average recall of all participants was 73.34% ± 5.99%, the FAR was 24.76% ± 6.37%, and the accuracy was 74.29% ± 5.99%.

### 3.3. Online System Performance

For each participant, after ten runs of online experiments, we obtained a total of 120 trials. The average results are listed in Table 2. It was seen that the average recall was 90.31% ± 3.86%, suggesting that the system was highly effective in capturing relevant events without significant loss of information. The FAR was 12.70% ± 4.73%. This relatively low FAR indicated that the system maintained a good balance between sensitivity and specificity, minimizing the occurrence of false alarms. The accuracy was 88.81% ± 3.61% and the response time was 431.95 ± 95.85 ms. This response time was within an acceptable range for many UAV operations, ensuring that the system could provide timely alerts in an emergency. Although the performance of the online detection system was affected by different subjects, the accuracy of most subjects reached around 90%, and the FAR was below 15%, except for Subject 1. The results showed the feasibility of this online detection system in the future application of UAV emergency detection.

## 4. Conclusions

This paper studied the brain neural signatures of UAV operators in emergencies and built an EEG signals-based online detection system for UAV emergencies. We found regularity characteristics similar to classic ERP components like vMMN and CNV, probably related to the visual stimuli that do not match the prior memory and response preparation. Source analysis results showed that after the onset of emergencies, the occipital, parietal, temporal, and frontal lobe regions were activated successively, which were related to emotional processing, cognitive processing, and decision-making caused by visual stimuli. We obtained the model with the best classification effect through offline experiment, and then built the online detection system through this model. The online experiments indicated that the system achieved an accuracy rate of over 88% in identifying emergencies with a response time of 431.95 ms from the emergency onset. This research contributes to the comprehension of operators’ brain activity in emergencies and advances the development of an EEG-based emergency detection approach to assist UAV operations.

## Figures and Tables

**Table 1:** Offline classification performance.

Subject	Accuracy	Recall	FAR
1	73.42%	70.33%	23.50%
2	72.05%	69.75%	25.65%
3	60.84%	60.22%	38.52%
4	75.11%	76.00%	25.77%
5	80.74%	82.11%	20.63%
6	78.35%	75.21%	18.51%
7	73.12%	74.84%	28.60%
8	80.67%	78.28%	16.93%
Mean ±	74.29%	73.34%	24.76%
Std	5.99%	6.20%	6.37%

This table displays the accuracy of the HDCA model, which is the best-performing model, for each individual subject.

**Table 2:** Online detection system performance.

Subject	Accuracy	Recall	FAR	Reponse Time
1	79.38%	82.50%	23.73%	674.75 ms
2	91.41%	91.67%	8.85%	452.73 ms
3	90.08%	94.58%	14.43%	363.88 ms
4	90.29%	95.42%	14.84%	392.14 ms
5	89.17%	87.50%	9.17%	368.57 ms
6	89.89%	90.42%	10.65%	398.16 ms
7	90.06%	88.75%	8.63%	425.35 ms
8	90.20%	91.67%	11.27%	380.00 ms
Mean ±	88.81%	90.31%	12.70%	431.95 ms
Std	3.61%	3.86%	4.73%	95.85 ms

This table presents the average accuracy for each individual subject using the HDCA method, which is the best-performing model, as detailed above.

**Figure A1:**
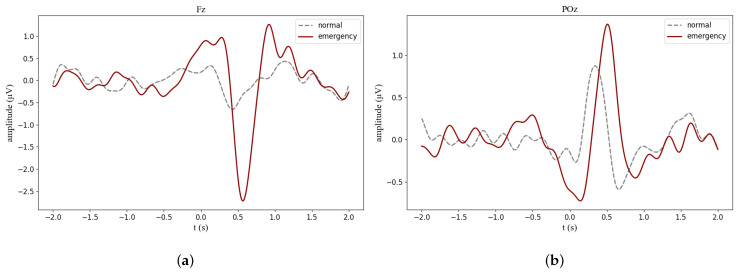
Average ERP from −2 s to 2 s of Subject 1. (**a**,**b**) represent the ERPs at electrodes Fz and POz, respectively.

**Figure A2:**
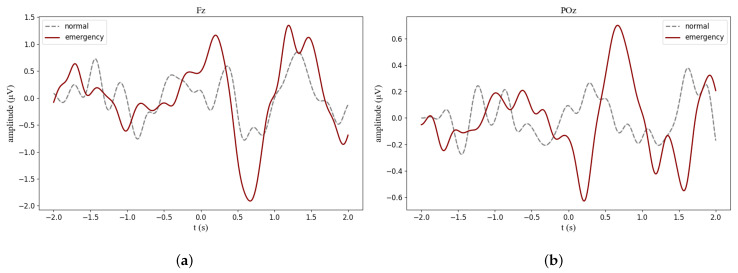
Average ERP from −2 s to 2 s of Subject 2. (**a**,**b**) represent the ERPs at electrodes Fz and POz, respectively.

**Figure A3:**
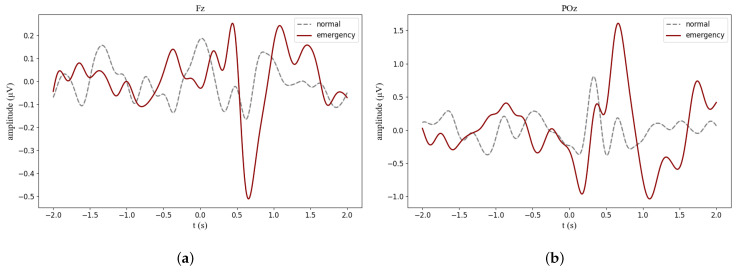
Average ERP from −2 s to 2 s of Subject 3. (**a**,**b**) represent the ERPs at electrodes Fz and POz, respectively.

**Figure A4:**
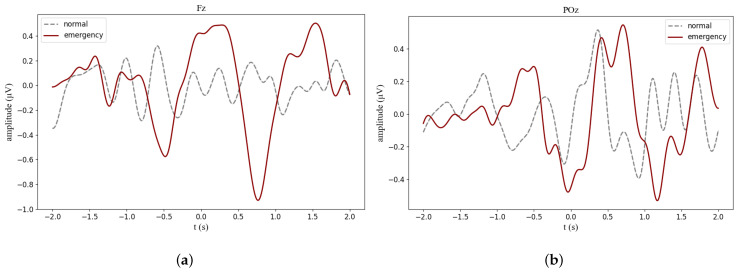
Average ERP from −2 s to 2 s of Subject 4. (**a**,**b**) represent the ERPs at electrodes Fz and POz, respectively.

**Figure A5:**
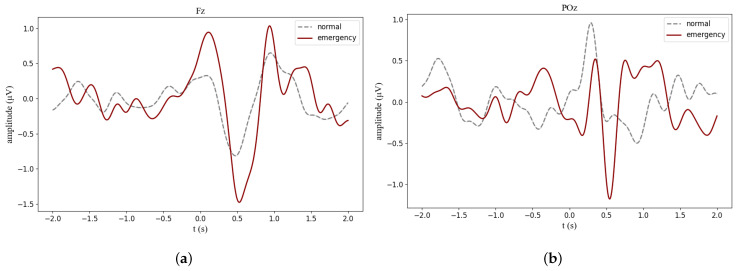
Average ERP from −2 s to 2 s of Subject 5. (**a**,**b**) represent the ERPs at electrodes Fz and POz, respectively.

**Figure A6:**
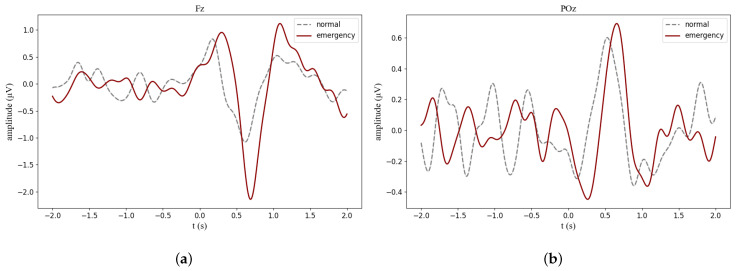
Average ERP from −2 s to 2 s of Subject 6. (**a**,**b**) represent the ERPs at electrodes Fz and POz, respectively.

**Figure A7:**
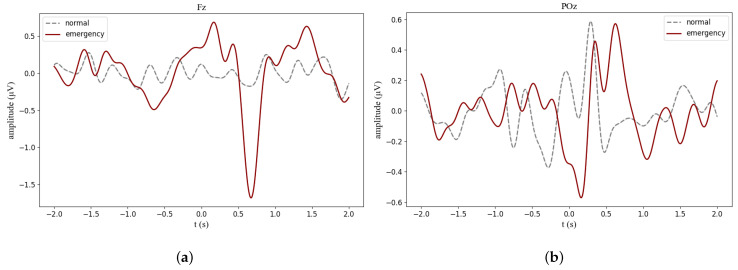
Average ERP from −2 s to 2 s of Subject 7. (**a**,**b**) represent the ERPs at electrodes Fz and POz, respectively.

**Figure A8:**
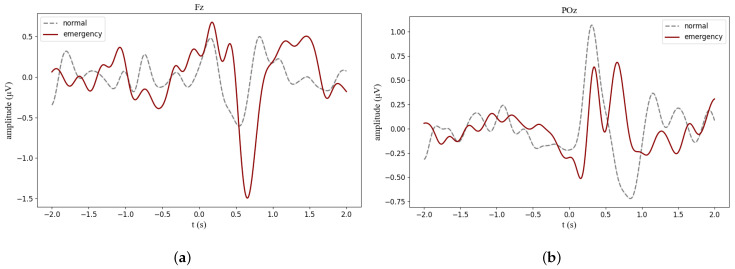
Average ERP from −2 s to 2 s of Subject 8. (**a**,**b**) represent the ERPs at electrodes Fz and POz, respectively.

**Figure A9:**
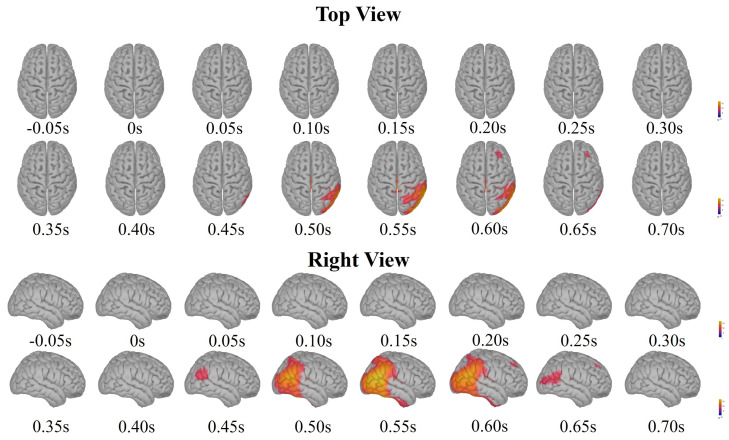
The grand-averaged activation pattern of Subject 2 in the source space. The source-imaging results are displayed with the time interval of 100 ms. The source-space activity is imaged from two projection angles: top view and right view.

**Figure A10:**
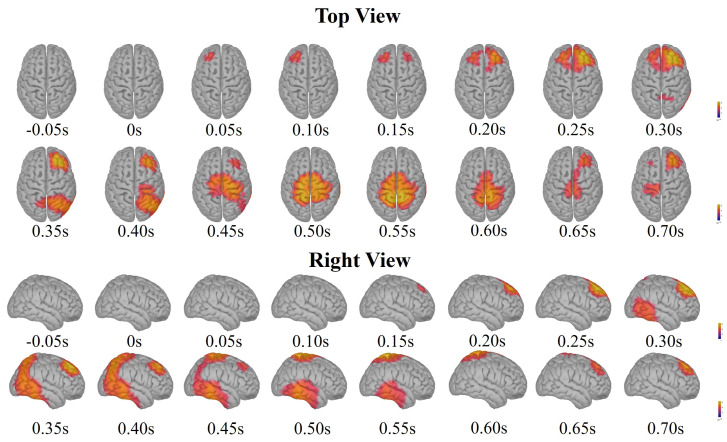
The grand-averaged activation pattern of Subject 3 in the source space. The source imaging results are displayed with the time interval of 100 ms. The source-space activity is imaged from two projection angles: top view and right view.

**Figure A11:**
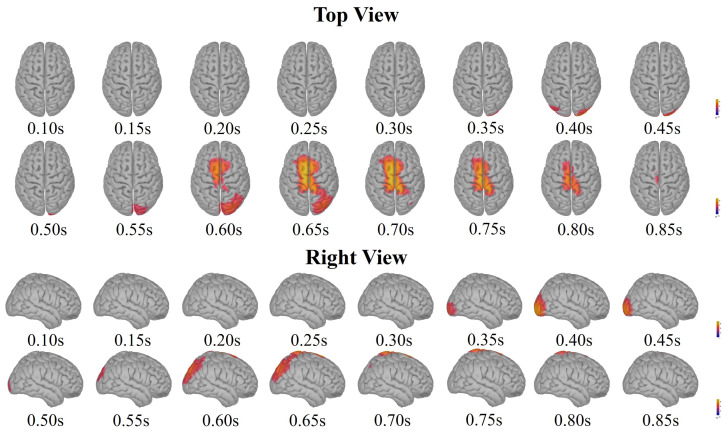
The grand-averaged activation pattern of Subject 4 in the source space. The source imaging results are displayed with the time interval of 100 ms. The source-space activity is imaged from two projection angles: top view and right view.

**Figure A12:**
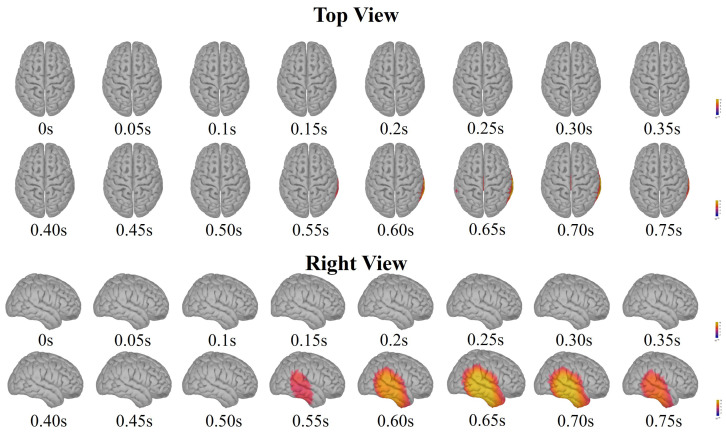
The grand-averaged activation pattern of Subject 5 in the source space. The source imaging results are displayed with the time interval of 100 ms. The source-space activity is imaged from two projection angles: top view and right view.

**Figure A13:**
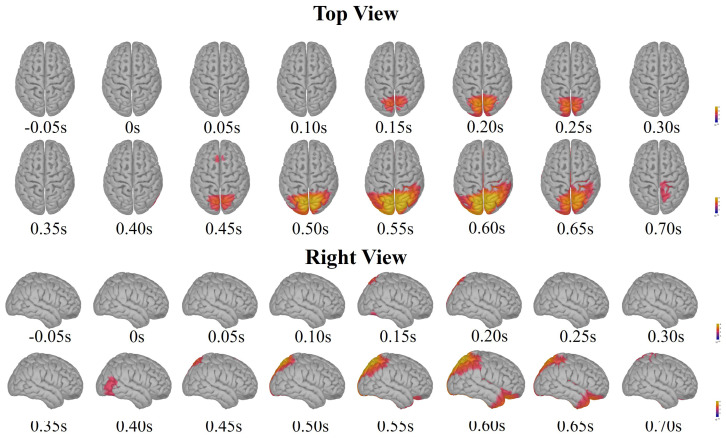
The grand-averaged activation pattern of Subject 6 in the source space. The source imaging results are displayed with the time interval of 100 ms. The source-space activity is imaged from two projection angles: top view and right view.

**Figure A14:**
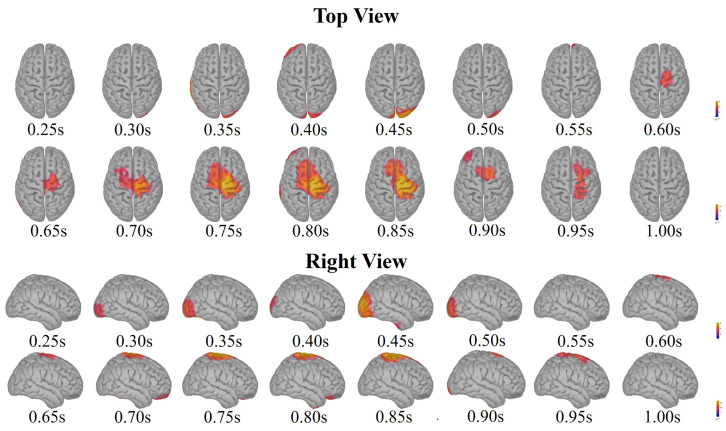
The grand-averaged activation pattern of Subject 7 in the source space. The source imaging results are displayed with the time interval of 100 ms. The source-space activity is imaged from two projection angles: top view and right view.

**Figure A15:**
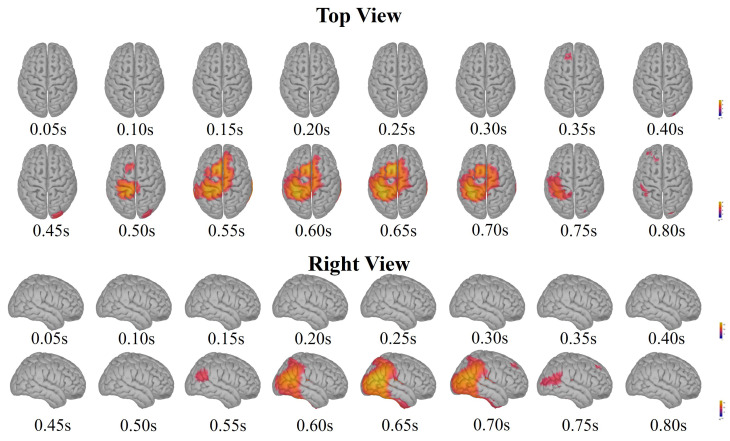
The grand-averaged activation pattern of Subject 8 in the source space. The source imaging results are displayed with the time interval of 100 ms. The source-space activity is imaged from two projection angles: top view and right view.

## Data Availability

The data presented in this study are available on request from the corresponding author.
